# How the coronavirus pandemic has affected the clinical management of Philadelphia-negative chronic myeloproliferative neoplasms in Italy—a GIMEMA MPN WP survey

**DOI:** 10.1038/s41375-020-0953-3

**Published:** 2020-07-03

**Authors:** Francesca Palandri, Alfonso Piciocchi, Valerio De Stefano, Massimo Breccia, Guido Finazzi, Alessandra Iurlo, Paola Fazi, Stefano Soddu, Bruno Martino, Sergio Siragusa, Francesco Albano, Francesco Passamonti, Marco Vignetti, Alessandro M. Vannucchi

**Affiliations:** 1 Institute of Hematology “L. and A. Seràgnoli”, Sant’Orsola-Malpighi University Hospital, Bologna, Italy; 2 GIMEMA Data Center, Fondazione GIMEMA Franco Mandelli Onlus, Rome, Italy; 3 Department of Radiological and Hematological Sciences, Section of Hematology, Catholic University and Fondazione Policlinico A. Gemelli IRCCS, Rome, Italy; 4 Department of Precision and Translational Medicine, Sapienza University, Rome, Italy; 5 Hematology and Bone Marrow Transplant Unit, ASST Papa Giovanni XXIII, Bergamo, Italy; 6 Division of Hematology, Foundation IRCCS Ca’ Granda—Ospedale Maggiore Policlinico, Milan, Italy; 7 Division of Hematology, Azienda Ospedaliera ‘Bianchi Melacrino Morelli’, Reggio Calabria, Italy; 8 Hematology Unit, Department “PROMISE”, University of Palermo, Palermo, Italy; 9 Department of Emergency and Organ Transplantation (D.E.T.O.), Hematology Section, University of Bari, Bari, Italy; 10 Division of Hematology, Department of Medicine and Surgery, Ospedale di Circolo, ASST Sette Laghi, University of Insubria, Varese, Italy; 11 CRIMM, Center Research and Innovation of Myeloproliferative Neoplasms, AOU Careggi, University of Florence, Florence, Italy

**Keywords:** Myeloproliferative disease, Quality of life

## To the Editor:

We read with interest the perspective by von Lilinfeld-Toal et al. that provides recommendations for the management of cancer patients during the coronavirus disease 2019 (COVID-19) pandemic, caused by the spreading of the coronavirus SARS-CoV-2 [1]. In asymptomatic patients treated with tyrosine kinase inhibitors, the EHA Infectious Disease Scientific Working Group recommended continuing the therapy unmodified. In case of severe COVID-19, the Authors would consider the *JAK1/2* inhibitor (JAKi) ruxolitinib as therapy for hyperinflammation. By *JAK1* inhibition, ruxolitinib had demonstrated to exert a broad anti-inflammatory activity against the myeloproliferative neoplasms (MPNs) cytokine storm and has been used in the setting of COVID-19 infection with positive results [2]. Conversely, ruxolitinib may affect the immune response by different effects on immune cells, including inhibition of differentiation, function, and migration of dendritic cells, reduced in vivo T-cell (regulatory, Th1 and Th17) frequency and cytokine production, inhibition of NK cells killing activity, proliferation, and cytokine production [3].

Philadelphia-negative MPNs include polycythemia vera (PV), essential thrombocythemia (ET), and myelofibrosis (MF) [4] and are characterized by increased thrombotic risk, progressive splenomegaly/symptoms, and reduced survival [5]. In MF and PV, infections represent a frequent complication, due to disease-related factors and use of ruxolitinib [6].

To understand how the behavior of Italian hematologists towards MPN has changed during the COVID-19 pandemic, and how ruxolitinib was managed, the GIMEMA (Gruppo Italiano Malattie EMatologiche dell’Adulto) MPN Working Party e‐mailed to 239 hematologists, belonging to 102 Italian hematology institutions, an anonymous online questionnaire (Supplementary Table) of 28 multiple choice questions. The survey was completed by 92 (38.5%) hematologists from 63 different Centers.

For MPN diagnosis, 93.5% of physicians continued to routinely assess *JAK2*, *MPL*, or *CALR* genotyping according to standard indications. During the pandemic, marrow biopsy was performed by 73.9% of respondents normally, by 10.9% only in the suspect of MF, and never by 14.1%.

In PV patients, 65.2% of respondents prescribed phlebotomies with a hematocrit (HCT) target at ≤45%, while 32.6% accepted the target HCT to >48%, and 2.2% did not suggest phlebotomies at all during the pandemic.

**Fig. 1:**
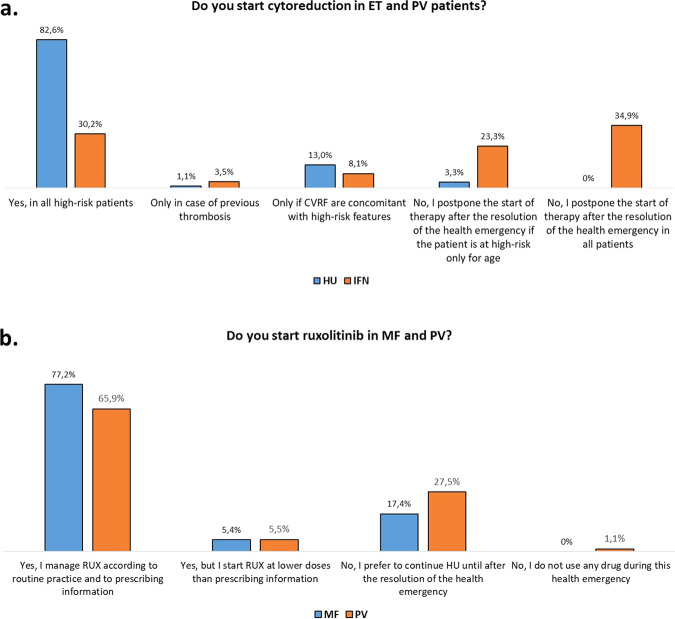
Start of cytoreductive therapies in ET and PV patients (**a**) and start of ruxolitinib in MF and PV patients during the COVID-19 pandemic (**b**). HU hydroxyurea, IFN interferon, ET essential thrombocythemia, PV polycythemia vera, MF myelofibrosis. Survey data were collected and managed using the REDCap electronic data capture tools hosted at the GIMEMA Foundation. Most responders have >10 years of clinical experience on MPNs and >20 patients in annual follow-up for each disease. However, only 10.9% of the clinicians directly followed MPN patients affected by COVID-19.

Hydroxyurea (HU) was started in all ET and PV patients at high thrombotic risk by 82.6% of hematologists; however, 13% started HU only if cardiovascular risk factors were concomitant to high-risk features. Conversely, >50% of the hematologists declared to postpone interferon (IFN) after the resolution of the pandemic (Fig. 1a). Instead, therapies already in place were not modified by the pandemic, with 93.5% and 88.8% of clinicians managing HU and IFN, respectively, according to routine practice. Only 2.2 and 5.6% of the hematologists suggested to discontinue HU or IFN, while 4.3 and 5.6% decreased their doses.

**Fig. 2:**
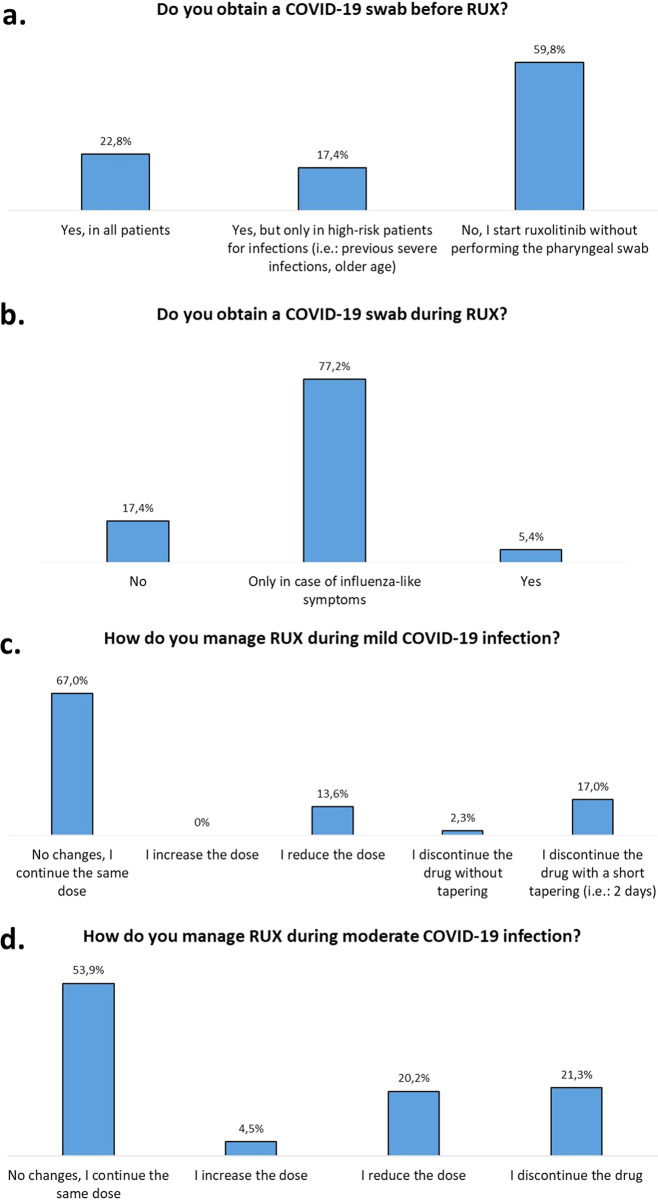
Management of COVID-19 screening before (**a**) or during (**b**) ruxolitinib and management of ruxolitinib in case of mild (**c**) or moderate (**d**) COVID-19 infection. Mild infection: respiratory symptoms not requiring hospitalization. Moderate infection: hypoxia (SPO2 ≤94%) requiring ventilatory support but not mechanical ventilation.

The start of ruxolitinib was postponed in 17.4% and 28.6% of MF and PV patients, respectively. (Fig. 1b). Before ruxolitinib start, 40.2% of the hematologists obtained a negative COVID-19 pharyngeal swab (Fig. 2a), while only 5.4% required a negative pharyngeal swab during ruxolitinib (Fig. 2b). For 79.8% of respondents, ruxolitinib has no negative effect on COVID-19 infection, for 10.1% a negative influence may be restricted to patients with MF and/or a great disease burden, while for 10.1% a negative effect may always be anticipated. In case of mild and moderate COVID-19 infection, 67% and 58.4% of respondents did not change therapy, respectively (Fig. 2c, d).

In MF patients with an allogeneic transplant already scheduled, only 8.8% of hematologists proceeded without delay, while 15.4% postponed the transplant to pandemic resolution.

Regarding the use of phone contacts in place of in-person hematological visits, 12% of the hematologists believed that MF patients always require a full medical visit; this percentage decreases to 1.1% and to 0% when PV and ET were considered, respectively. Accordingly, 19.8%, 38%, and 50% of respondents converted >80% medical visits into phone follow-up in MF, PV, and ET patients, respectively. After the resolution of the pandemic, 67.3% of hematologists will implement the use of telemedicine (Supplementary Figure).

Notably, we documented no substantial difference in practice in colleagues with more (>10) years of experience, or who followed COVID-19-positive patients, or who work in the northern Italian regions most affected by the pandemic. This remarkable uniformity is probably owed to homogeneous recommendations from the central government and from Italian Societies of Hematology and Transplantation.

Diagnostic procedures remained consistent to standard criteria, with most patients receiving molecular and histology evaluations during the pandemic, as already described in Italy [4, 5, 7, 8]. The therapeutic approach was also adherent to international guidelines, with phlebotomies for HCT > 45%, initiation of cytoreduction in patients at high thrombotic risk, and no treatment adjustment in most patients. However, about 1/3 of hematologists skipped or decreased the frequency of phlebotomies: any consequent increase in the thrombosis rate will have to be assessed in the future.

Concerning ruxolitinib, the ASH panel of MPN experts suggests not starting ruxolitinib during the pandemic, but, maintaining the drug if beneficial, with a slow tapering in case of discontinuation [9]. We conversely observed that ruxolitinib was started in most symptomatic patients, indicating that the potential clinical benefit outweighed the concern about ruxolitinib-related immunosuppression, particularly in MF. Accordingly, only 10.1% of hematologists believed that ruxolitinib may exacerbate the outcome of COVID-19 infection and a minority obtained pharyngeal swabs in asymptomatic patients.

The evaluation of each allogeneic transplant case individually well reflects the indications of the European Society for Blood and Marrow Transplantation to postpone transplant in low-risk patients, assessing on individual basis the risk-benefit ratio of transplant deferral [10].

Finally, telemedicine was perceived as an appropriate, though exceptional, follow-up strategy. While its future implementation in routine practice may possibly offer some benefits, some fundamental concerns need to be addressed regarding the challenges for proper patient management, mutual patient–doctor satisfaction, and legal protection.

Despite significant reduction of in-person visits, the clinical approach to MPN was only mildly modified during the pandemic. However, we acknowledge that clinical choices, particularly regarding the use of JAKi, may be based on multiple factors. Also, many clinicians were not directly involved in the treatment of COVID-19 MPN patients. Future epidemiological studies may clarify whether this is an underestimation or the result of appropriate patient management.

## Supplementary information

Supplemental Table

Supplemental Figure
