# Supplementary material for: How the coronavirus pandemic has affected the clinical management of Philadelphia-negative chronic myeloproliferative neoplasms in Italy—a GIMEMA MPN WP survey
Source: Leukemia. 2020 Jul 3;34(10):2805–8. doi: 10.1038/s41375-020-0953-3 (PMC7333222; doi:10.1038/s41375-020-0953-3)
Supplement: Supplementary file 1 — Supplemental Table [file 41375_2020_953_MOESM1_ESM.docx]

**Supplemental Table. Questionnaire and related answers**

| **Questions** | **Possible answers** | **Percentages** |
| --- | --- | --- |
| 1. **Where do you practice your clinical activity?** | 1. University | 43.5% |
|  | 1. Public hospital | 55.4% |
|  | 1. Private practice | 1.1% |
| 1. **How many years of experience do you have in the treatment of chronic myeloproliferative neoplasms?** | 1. < 5 | 9.9% |
|  | 1. 5-10 | 17.6% |
|  | 1. >10 | 72.5% |
| 1. **How many myelofibrosis (MF) patients do you see per year overall?** | 1. < 20 | 26.1% |
|  | 1. 20-100 | 52.2% |
|  | 1. >100 | 21.7% |
| 1. **How many polycythemia vera (PV) patients do you see per year overall?** | 1. < 20 | 10.9% |
|  | 1. 20-100 | 59.8% |
|  | 1. >100 | 29.3% |
| 1. **How many essential thrombocythemia (ET) patients do you see per year overall?** | 1. < 20 | 13.0% |
|  | 1. 20-100 | 51.1% |
|  | 1. >100 | 35.9% |
| 1. **Have you personally treated MPN-COVID patients?** | 1. Yes | 10.9% |
|  | 1. No | 89.1% |
| 1. **In a patient with a suspect of MPN, do you regularly perform the molecular test for driver mutations to confirm diagnosis?** | 1. Yes, in all MPNs | 93.5% |
|  | 1. Only in MF | 1.1% |
|  | 1. No, I postpone the molecular test after the resolution of the health emergency | 5.4% |
| 1. **In a patient with a suspect of MPN, do you regularly perform the bone marrow biopsy to confirm diagnosis** | 1. Yes, in all MPNs | 73.9% |
|  | 1. Only in triple-negative patients | 1.1% |
|  | 1. Only in the suspect of a MF | 10.9% |
|  | 1. No, I postpone the marrow biopsy after the resolution of the health emergency | 14.1% |
| 1. **In a patient with PV and hematocrit > 45%, do you schedule the phlebotomy?** | 1. Yes, immediately and in all patients | 65.2% |
|  | 1. Yes, but only if the haematocrit is > 48-50% | 32.6% |
|  | 1. No, I postpone phlebotomies until after the health emergency has been resolved in all patients | 2.2% |
| 1. **In a patient with PV or ET at high risk for thrombosis, do you promptly start cytoreductive therapy?** | 1. Yes, in all high-risk patients | 82.6% |
|  | 1. Only in case of previous thrombosis | 1.1% |
|  | 1. Only in presence of cardiovascular risk factors concomitant with older age and/or previous thrombosis | 13.0% |
|  | 1. No, I postpone the start of cytoreduction after the resolution of the health emergency if the patient is at high-risk only for age | 3.3% |
|  | 1. No, I postpone the start of cytoreduction after the resolution of the health emergency in all patients | 0% |
| 1. **In a patient with PV or ET at high risk for thrombosis, do you promptly start Interferon (IFN) therapy?** | 1. Yes, in all high-risk patients | 30.2% |
|  | 1. Only in case of previous thrombosis | 3.5% |
|  | 1. Only in presence of cardiovascular risk factors concomitant with older age and/or previous thrombosis | 8.1% |
|  | 1. No, I postpone the start of IFN after the resolution of the health emergency if the patient is at high-risk only for age | 23.3% |
|  | 1. No, I postpone the start of IFN after the resolution of the health emergency in all patients | 34.9% |
| 1. **In a patient with PV or ET under cytoreductive therapy (hydroxyurea or alkylating agents), how do you manage the therapy ?** | 1. I manage cytoreduction according to routine practice | 93.5% |
|  | 1. I decrease the dose of cytoreductive agents during the health emergency, to avoid potential immunosuppressive activity | 4.3% |
|  | 1. I discontinue cytoreduction if there is a good control of blood counts | 2.2% |
| 1. **In a patient with PV or ET under interferon (IFN) therapy, how do you manage the therapy?** | 1. I manage IFN according to routine practice | 88.8% |
|  | 1. I decrease IFN dose during the health emergency, to avoid potential immunosuppressive activity | 5.6% |
|  | 1. I discontinue IFN if there is a good control of blood counts | 5.6% |
| 1. **In a patient with symptomatic MF, do you start ruxolitinib?** | 1. Yes, I manage ruxolitinib according to routine practice and to prescribing information | 77.2% |
|  | 1. Yes, but I start ruxolitinib at lower doses than prescribing information | 5.4% |
|  | 1. No, I prefer to use hydroxyurea until after the resolution of the health emergency | 17.4% |
|  | 1. No, I do not use any drug during this health emergency | 0% |
| 1. **In a patient with symptomatic PV, resistant or intolerant to hydroxyurea, do you start ruxolitinib?** | 1. Yes, I manage ruxolitinib according to routine practice and to prescribing information | 65.9% |
|  | 1. Yes, but I start ruxolitinib at lower doses than prescribing information | 5.5% |
|  | 1. No, I prefer to continue hydroxyurea until after the resolution of the health emergency | 27.5% |
|  | 1. No, I do not use any drug during this health emergency | 1.1% |
| 1. **Before starting ruxolitinib therapy, do you require a covid-19 negative pharyngeal swab in a patient without symptoms** | 1. Yes, in all patients | 22.8% |
|  | 1. Yes, but only in high-risk patients for infections (i.e.: previous severe infections, older age) | 17.4% |
|  | 1. No, I start ruxolitinib without performing the pharyngeal swab | 59.8% |
| 1. **Do you obtain a covid pharyngeal swab in your patients under ruxolitinib therapy?** | 1. No | 17.4% |
|  | 1. Only in case of symptoms (fever, cough, etc etc) | 77.2% |
|  | 1. Yes, in all patients | 5.4% |
| 1. **In a patient with mild respiratory symptoms and a pharyngeal swab positive for covid-19, how do you manage ruxolitinib therapy?** | 1. No changes, I continue the same dose | 67.0% |
|  | 1. I reduce the dose | 13.6% |
|  | 1. I discontinue the drug without tapering | 2.3% |
|  | 1. I discontinue the drug with a short tapering (i.e.: 2 days) | 17.0% |
| 1. **In a patient with covid-19 infection requiring ventilatory support, but not mechanical ventilation, how do you manage ruxolitinib therapy?** | 1. No changes, I continue the same dose | 53.9% |
|  | 1. I reduce the dose | 20.2% |
|  | 1. I increase the dose | 4.5% |
|  | 1. I discontinue the drug | 21.3% |
| 1. **Do you believe that ruxolitinib may negatively influence the outcome of covid19-positive patients with PV or MF?** | 1. Yes, in all patients | 10.1% |
|  | 1. Yes, but only in MF patients | 4.5% |
|  | 1. Yes, but only in patients with a great burden of the disease | 5.6% |
|  | 1. No | 79.8% |
| 1. **In a patient with high-risk fit MF with a compatible donor, do you proceed for allogeneic transplantation?** | 1. Yes, in all patients | 8.8% |
|  | 1. I discuss each case individually with the Transplant Center | 75.8% |
|  | 1. No, I postpone the start of allogeneic transplant after the resolution of the health emergency in all patients | 15.4% |
| 1. **In patients with MF, do you replace hematological visits with telephone contacts?** | 1. Yes, in all cases | 3.3% |
|  | 1. Yes, unless there is an acute situation to handle | 84.8% |
|  | 1. No, MF patients require a full medical visit | 12.0% |
| 1. **What percentage of hematological visits have you converted into phone contacts with MF patients during this period?** | 1. 0-10% | 11.0% |
|  | 1. 10-50% | 33.0% |
|  | 1. 50-80% | 36.3% |
|  | 1. >80% | 19.8% |
| 1. **In patients with PV, do you replace hematological visits with telephone contacts?** | 1. Yes, in all cases | 5.4% |
|  | 1. Yes, unless there is an acute situation to handle | 93.5% |
|  | 1. No, PV patients require a full medical visit | 1.1% |
| 1. **What percentage of hematological visits have you converted into phone contacts with PV patients during this period?** | 1. 0-10% | 3.3% |
|  | 1. 10-50% | 25.0% |
|  | 1. 50-80% | 33.7% |
|  | 1. >80% | 38.0% |
| 1. **In patients with ET, do you replace hematological visits with telephone contacts?** | 1. Yes, in all cases | 7.7% |
|  | 1. Yes, unless there is an acute situation to handle | 92.3% |
| 1. **What percentage of hematological visits have you converted into phone contacts with ET patients during this period?** | 1. 0-10% | 3.3% |
|  | 1. 10-50% | 14.1% |
|  | 1. 50-80% | 32.6% |
|  | 1. >80% | 50.0% |
| 1. **Do you think that any change in your clinical behavior during the COVID-19 emergency will affect also the post-emergency phase?** | 1. No, I am going back to the previous standard | 32.6% |
|  | 1. Yes, I will use the phone/emails more and reduce the frequency of the hematological visits, but only for patients who are not on cytoreductive therapy | 20.7% |
|  | 1. Yes, I will use the phone/emails more and reduce the frequency of the hematological visits for patients that are not on cytoreductive therapy or that are on chronic cytoreductive therapy | 29.3% |
|  | 1. Yes, I will use the phone/emails more and reduce the frequency of the hematological visits in all patients, excluding only the patients on therapy with JAK-inhibitors | 17.4% |
