# Supplementary figures and images for: How the coronavirus pandemic has affected the clinical management of Philadelphia-negative chronic myeloproliferative neoplasms in Italy—a GIMEMA MPN WP survey
Source: Leukemia. 2020 Jul 3;34(10):2805–8. doi: 10.1038/s41375-020-0953-3 (PMC7333222; doi:10.1038/s41375-020-0953-3)

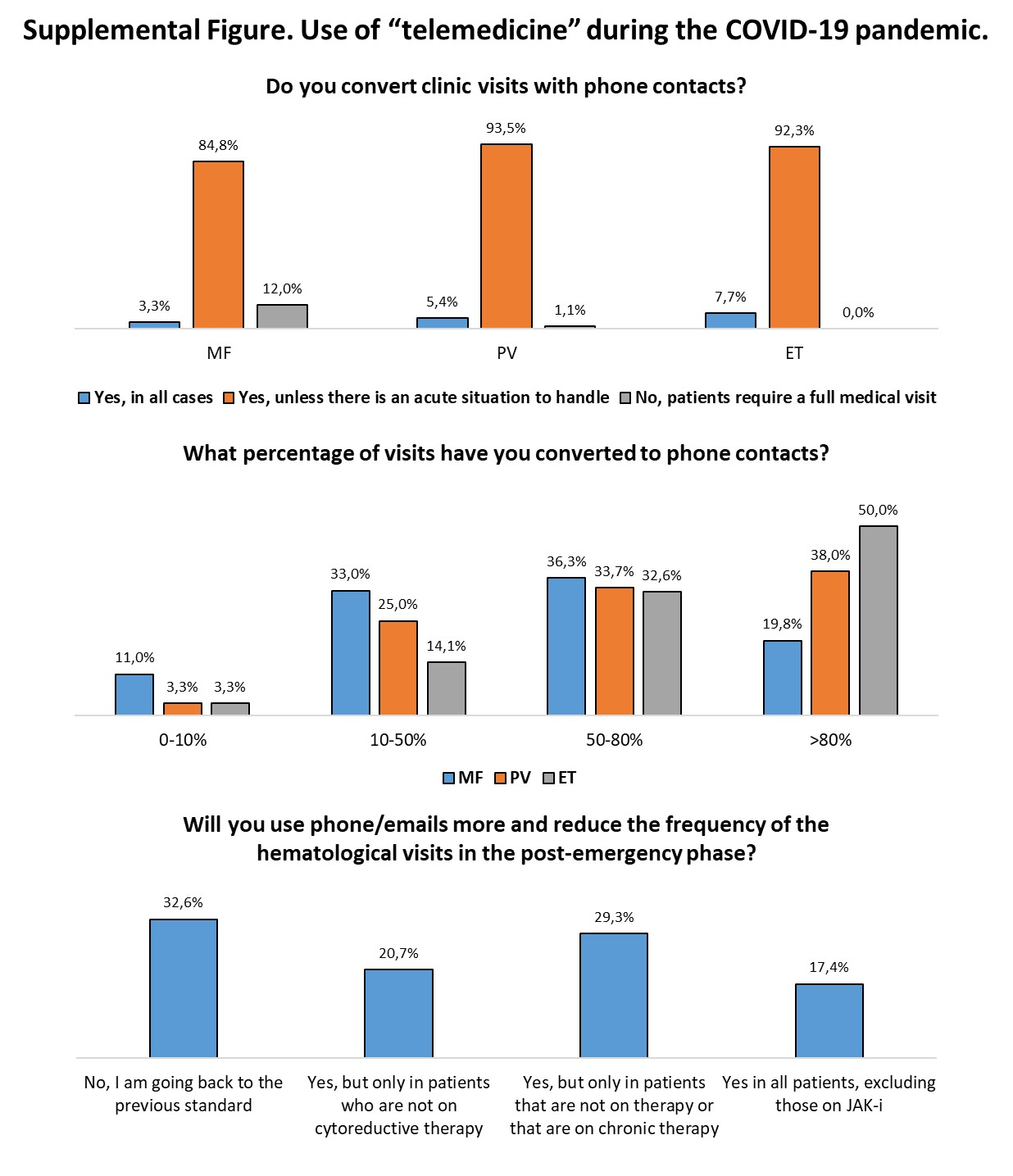

Supplement: Supplementary file 2 — Supplemental Figure [file 41375_2020_953_MOESM2_ESM.jpg]
